# Local expression of tumor necrosis factor-receptor 1:immunoglobulin G can induce salivary gland dysfunction in a murine model of Sjögren's syndrome

**DOI:** 10.1186/ar2888

**Published:** 2009-12-14

**Authors:** Jelle L Vosters, Hongen Yin, Nienke Roescher, Marc R Kok, Paul P Tak, John A Chiorini

**Affiliations:** 1 Molecular Physiology and Therapeutics Branch, National Institute of Dental and Craniofacial Research, National Institutes of Health, 10 Center Drive, Bethesda, MD 20892, USA; 2 Division of Clinical Immunology and Rheumatology, Academic Medical Center, University of Amsterdam, Meibergdreef 9, 1100 DD, Amsterdam, The Netherlands

## Abstract

**Introduction:**

Tumor necrosis factor is a pleiotropic cytokine with potent immune regulatory functions. Although tumor necrosis factor inhibitors have demonstrated great utility in treating other autoimmune diseases, such as rheumatoid arthritis, there are conflicting results in Sjögren's syndrome. The aim of this study was to assess the effect of a locally expressed tumor necrosis factor inhibitor on the salivary gland function and histopathology in an animal model of Sjögren's syndrome.

**Methods:**

Using in vivo adeno associated viral gene transfer, we have stably expressed soluble tumor necrosis factor-receptor 1-Fc fusion protein locally in the salivary glands in the Non Obese Diabetic model of Sjögren's syndrome. Pilocarpine stimulated saliva flow was measured to address the salivary gland function and salivary glands were analyzed for focus score and cytokine profiles. Additionally, cytokines and autoantibody levels were measured in plasma.

**Results:**

Local expression of tumor necrosis factor-receptor 1:immunoglobulin G fusion protein resulted in decreased saliva flow over time. While no change in lymphocytic infiltrates or autoantibody levels was detected, statistically significant increased levels of tumor growth factor-β1 and decreased levels of interleukin-5, interleukin-12p70 and interleukin -17 were detected in the salivary glands. In contrast, plasma levels showed significantly decreased levels of tumor growth factor-β1 and increased levels of interleukin-4, interferon-γ, interleukin-10 and interleukin-12p70.

**Conclusions:**

Our findings suggest that expression of tumor necrosis factor inhibitors in the salivary gland can have a negative effect on salivary gland function and that other cytokines should be explored as points for therapeutic intervention in Sjögren's syndrome.

## Introduction

Sjögren's syndrome (SS) is a systemic autoimmune disorder affecting secretory tissue, including the lacrimal and salivary glands (SGs), resulting in keratoconjunctivitis sicca and xerostomia. SS is characterized by mononuclear cell infiltrates in the salivary and lacrimal glands as well as the presence of autoantibodies in serum. Other organ systems may be involved as well and around 5% of the patients develop B cell lymphoma [1,2]. There is still an unmet need for an effective treatment of SS.

Anti-tumor necrosis factor (TNF) therapies have been widely and successfully used several chronic autoimmune diseases, such as rheumatoid arthritis (RA) and Crohn's disease. Clinical trials with anti-TNF antibodies and etanercept showed improvement in 60 to 70% of the RA patients [3,4]. Patients with SS have been reported to have elevated serum pro-inflammatory cytokine levels compared with normal volunteers [5,6] and TNF is also overexpressed in the SGs of SS patients [7]. However, the use of anti-TNF agents in patients with the autoimmune disease SS has shown conflicting results [8,9]. Beneficial results were shown in an open study, while inefficacy of anti-TNF was shown in a randomized, double-blind, placebo-controlled trial.

TNF promotes inflammation by stimulating and inducing other inflammatory cytokines and adhesion molecules and is a key player in the cytokine balance [4]. In contrast, TNF can also exhibit anti-inflammatory activities, for instance by blocking the development of autoreactive T cells [10]. Moreover, adoptive transfer of ex vivo TNF treated splenocytes from autoimmune diabetic female non-obese diabetic (NOD) mice into irradiated pre-diabetic male mice prevented the development of hyperglycemia in 80% of the recipients. Recently, a T-cell based mechanism has been proposed to explain the dual effect of anti-TNF therapy in the treatment of autoimmune diseases in which TNF can function as a pro-inflammatory cytokine as well as an anti-inflammatory immunoregulatory molecule by altering the balance of regulatory T cells [11].

The National Institute of Dental and Craniofacial Research (NIDCR) Sjögren's clinic has previously investigated the efficacy of systemic etanercept treatment in SS patients and could not demonstrate clinical benefit [12]. Follow-up studies of cytokine levels in these patients before and after treatment revealed no decrease in TNF and other pro-inflammatory cytokines [13,14]. The reasons for the failed clinical trials are not well understood, but it is conceivable that the effects would be different if a more localized approach was used. Gene therapy offers the possibility to engineer cells to express therapeutic proteins locally at high levels. Previously, we reported successful gene transfer of interleukin (IL)-10 and vasoactive intestinal peptide (VIP) to mouse SGs [15,16]. To investigate the effects of local TNF blockade using gene therapy, we evaluated the effect of a locally expressed TNF inhibitor on the SG function and histopathology in the NOD model of SS.

## Materials and methods

### Cell lines

Human embryonic kidney 293T cells were grown in DMEM (Invitrogen, Carlsbad, CA, USA). This medium was supplemented with 10% heat-inactivated fetal bovine serum (FBS, Life Technologies, Rockville, MD, USA), 2 mM L-glutamine, penicilline (100 U/ml), and streptomycin (100 μg/ml; Biofluids, Rockville, MD, USA) as previously described [15]. Human fibrosarcoma (WEHI) cells were grown in RPMI 1640 (Invitrogen). This medium was supplemented with 10% FBS, 2 mM L-glutamine, penicilline (100 U/ml) and streptomycin (100 μg/ml), gentamycin (10 mg/ml; Invitrogen) and 1 M hepes (Invitrogen).

### Construction, expression and biological activity of plasmid

We previously reported the construction of recombinant Adeno Associated Virus (rAAV)-β galactosidase (rAAV2-LacZ) encoding β-galactosidase [17]. In this study we used the extra-cellular domain of human 55 kDa Tumor Necrosis Factor Receptor type 1 (hTNFR1; p55) coupled to the Fc-part of mouse Immunoglobulin G1 (IgG1), kindly provided by Dr J. Kolls [18]. This gene was cloned into the rAAV plasmid containing a Cytomegalovirus (CMV) promoter and the Inverted Terminal Repeat (ITRs) sequences for AAV serotype 2 (AAV2). The resulting plasmid (pAAV2-CMV-hTNFR1-mIgG1) was transfected into 293T cells and secretion of the protein in the supernatant was measured by western blotting and an ELISA-kit for hTNFR1 (R&D systems, Minneapolis, MN, USA). The biological activity was measured in an assay using WEHI fibrosarcoma cells. This assay is based on the cytotoxic effect of TNF-α on WEHI and the neutralizing effect of soluble hTNFR1 on bioactive TNF [19].

### Vector production

To generate rAAV serotype 2 vectors (rAAV2), we used the adenoviral helper packaging plasmid pDG. Plates (15 cm) of approximately 40% confluent 293 T cells were cotransfected with either pAAV-LacZ, pAAV-TNFR1 or pAAV-Luc and pDG according to standardized methods [20]. Clarified cell lysates were adjusted to a refractive index of 1.372 by addition of Cesium Chloride (CsCl) and centrifuged at 38,000 rpm for 65 hr at 20°C [21]. Equilibrium density gradients were fractionated and fractions with a refractive index of 1.369 to 1.375 were collected. The titer of DNA physical particles in rAAV stocks was determined by Quantitative-Polymerase Chain Reaction (Q-PCRand the vectors were stored at -80°C. On the day of vector administration to NOD mice, the vector was dialyzed for 3 hr against saline.

### Animals

Female NOD mice used in this study were obtained from the University of Florida College of Medicine, Department of Pathology, Immunology and Laboratory Medicine. All the mice were bred and maintained under specific pathogen free conditions in the animal facilities of the National Institute of Molecular Physiology and Therapeutics Branch (MPTB). All the animals were kept under specific conditions as described in Table 1. Animal protocols were approved by MPTB Animal Care and Use Committee and the National Institutes of Health (NIH) Biosafety Committee. All mice received water and food ad libitum. Blood glucose levels were measured by tail cut once a week starting at 12 weeks of age, using a OneTouch monitor (LifeScan, Milpitas, CA, USA). Mice with blood glucose levels >400 mg/dl were treated by subcutaneous injection (1 U/24 h) with long acting Humalin N (Eli Lilly, Indianapolis, IN, USA). The blood sugar level was monitored previously in other studies with NOD mice and does not appear to affect their behavior, feeding activity, or SG activity compared with non-diabetic NOD mice [15].

**Table 1:** Specific conditions for NOD mice

**Food**	Teklad Global 18% Protein Rodent Diet (2018S), Harlan
**Water**	Autoclaved
**Bedding**	Care fresh (non-bleached)
**Day/Night light cycle**	14/10 hours
**Racks**	Vented heap-filtered microisolator
**Cage changing**	1/week
**Cleaning agents**	Clidox 1:18:1
**Cage set up**	Autoclaved
**Helicobacter**	Tested negative
**MPV**	Tested negative
**MVM**	Tested negative
**MNV**	Tested negative

MPV = mouse parvovirus, MVM = minute virus of mice, MNV = mouse norovirus.

### rAAV2 vector administration and plasma/saliva collection

Vectors were delivered into the submandibular glands by retrograde instillation as previously described [15]. Briefly, mild anesthesia was induced by ketamine (100 mg/mL, 1 mL/kg body weight (BW); Fort Dodge Animal Health, Fort Dodge, IA, USA) and xylazine (20 mg/mL, 0.7 mL/kg body weight; Phoenix Scientific, St. Joseph, MO, USA) solution given intramuscularly (im). Ten minutes after im injection of atropine (0.5 mg/kg BW; Sigma, St. Louis, MO, USA), female NOD mice at the age of eight weeks were administered 50 μl vector into both submandibular glands by retrograde ductal instillation (1 × 10^10 ^particles/gland) using a thin cannula (Intermedic PE10, Clay Adams, Parsippany, NJ, USA). The vector dose was chosen based on previously published results, which showed detectable transgene activity above 10^9 ^particles/gland [15]. Saliva collection was done at several time points: baseline (six weeks of age), 16, 20 and 24 weeks of age. Mice were anesthetized as described above and saliva secretion was induced by subcutaneous (sc) injection of pilocarpine (0.5 mg/kg BW; Sigma-Aldrich, St. Louis, MO, USA). Stimulated whole saliva was gravimetrically collected for 20 minutes from the oral cavity with a hematocrit tube (Drummond Scientific Company, Broomall, PA, USA) placed into a preweighed 0.5 ml microcentrifuge tube, and the volume was determined by weight as previously described [15]. The presented saliva data are the result of two independent experiments (N = 20 for LacZ and N = 19 for TNFR1:IgG). Blood was collected at the saliva collection time points by retro-orbital plexus bleeding, from which plasma was separated by centrifugation for five minutes in an eppendorf tube centrifuge. Plasma was stored at -80°C until further analysis.

### Histologic assessment of submandibular glands

Submandibular glands were removed for histologic analysis from NOD mice at the time of sacrifice, 24 weeks of age, and placed overnight in 10% formalin. After fixation, the tissues were dehydrated in ethanol series and embedded in paraffin according to standard techniques. Sections were cut at 5 μm with 50 μm between consecutive sections and subsequently stained with hematoxylin and eosin (H&E). Histopathologic scoring was performed using the focus score system in which a focus is defined as an aggregate of 50 or more lymphocytes. A total of three sections per SG were scored and the results were calculated and expressed as foci per 4 mm^2^. The focus scores were assessed blindly by three different examiners (JV, HY, NR) and the mean scores were determined.

### Immunohistochemistry

Infiltrating lymphocytic cells were phenotyped on frozen SG sections by immunohistochemistry with antibodies directed towards CD4 (clone L3T4, eBioscience, San Diego, CA, USA), CD8 (clone 53-6.7, eBioscience), CD19 (clone ID3, BD, Breda, The Netherlands), and CD138 (clone 281-2, BD, Breda).

Sections were blocked with endogenous peroxidase with 1% H_2_O_2_. Aspecific binding was reduced by blocking with PBS (phosphate buffered saline)/1% BSA (Bovine Serum Albumin) + 10% goat serum for 30 minutes at room temperature (RT). After blocking, the sections were incubated with primary antibody at 4°C overnight (O/N). Stainings were visualised with a horseradish peroxidise (HRP)-labelled goat-anti-rat secondary antibody (Southern Biotechnology, Birmingham, AL, USA) and developed by AEC substrate (Dako, Glostrup, Denmark). Concentration- and isotype-matched control antibodies were included as negative control. All sections were coded and randomly analyzed by computer-assisted image analysis. For all markers, 18 high-power fields were analyzed. The images of the high-power fields were analyzed using the Qwin analysis system (Leica, Cambridge, UK), as described previously [22]. Positive staining of the cellular markers was expressed as the number of positive cells/mm^2^.

### Quantification of cytokine profiles

The levels of several cytokines were determined after extraction of soluble protein from the SGs. After measuring the wet weight, SGs were homogenized in protease buffer PBS and complete protease inhibitor cocktail (Roche Applied Science, Indianapolis, IN, USA). The excessive connective tissue and large aggregate debris were removed by 15 minutes centrifugation at 1500 × g. Total protein in the supernatant was determined with BCA™ protein assay kit (Pierce, Rockford, IL, USA) according to the manufacturer's instructions. Human sTNFR1 was measured by a commercial available ELISA kit (R&D Duokit, Minneapolis, MN, USA). mIL-4, mIL-5, mIL-6, mIL-10, mIL-12p70, mIL-17, MCP-1, mTGF-β1, mIFN-γ and mTNF-α were measured commercially using SearchLight proteome assay (Pierce Biotechnology, Woburn, MA, USA). This assay is a multiplexed sandwich ELISA procedure for detecting multiple cytokines in the same minimal sample. The same analytes were determined in plasma samples. Lower detection limits for this assay are: mIL-4: 1.2 pg/ml, mIL-5: 2.3 pg/ml, mIL-6: 5.5 pg/ml, mIL-10: 1.6 pg/ml, mIL-12p70: 0.78 pg/ml, mIL-17: 1.6 pg/ml, MCP-1: 0.78 pg/ml, mTGF-β1: 6.8 pg/ml, mIFN-γ: 7.8 pg/ml, mTNF-α: 3.1 pg/ml.

### Determination of autoantibodies

Plasma samples of 24 wk old NOD mice were analyzed for autoantibodies against nuclear antigens (ANA), Sjögren's syndrome (SS) A/Ro and SSB/La. ANA (total Ig), the autoantibody against SSA/Ro (total Ig) and SSB/La (total Ig) were measured by a commercial available ELISA kit (Alpha Diagnostic International, San Antonio, TX, USA) according the manufacturer's protocol.

### Statistical analysis

Differences in salivary flow, cytokines and lymphocytic cell type characterization among experimental groups were assessed using the non-parametric Wilcoxon's ranksum test. The focus scores and autoantibodies were assessed using unpaired Student's t-test to compare differences between groups. Non-parametric correlations were assessed using Spearman's Rho test and was performed with SPSS for Windows (SPSS version 16.0.02, Chicago, IL, USA) and all the other analyses were performed with GraphPad Prism statistical software (GraphPad Software Inc. version 4.02, La Jolla, CA, USA) using a *P *value less than 0.05 as statistically significant.

## Results

### Biological activity of TNFR1:IgG

**Figure 1:**
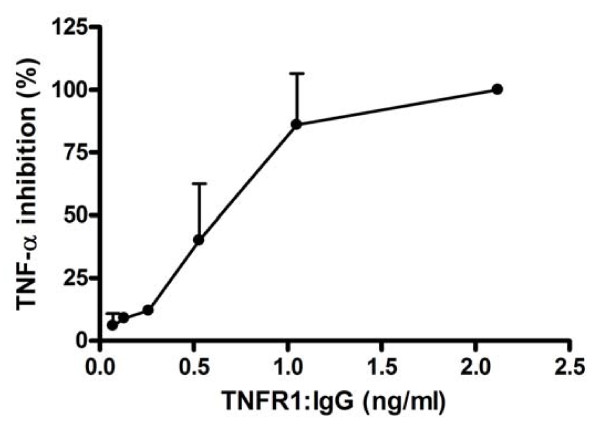
**In vitro biological activity assay for TNFR1:IgG**. The biological activity of the TNFR1:IgG was determined by adding supernate from TNFR1:IgG expressing cells to WEHI fibrosarcoma cells and measuring its ability to block TNF induced apoptosis in these cells. Increasing concentrations of TNFR1:IgG resulted in inhibiting TNF (62.5 pg/ml) induced apoptosis of WEHI cells. Data shown are mean ± SD (N = 2 independent experiments).

Prior to stable expression of TNFR1:IgG in the SGs of NOD mice, we confirmed that the fusion protein was biologically active in blocking TNF activity in vitro. To test for biologic activity of TNFR1:IgG, dilutions of supernatant from TNFR1:IgG expressing cells were preincubated with 62.5 pg/ml hTNF, this mixture was added to WEHI cells (a TNF sensitive cell line) overnight, and cell death was measured. In the absence of TNFR1:IgG, 62.5 pg/ml hTNF was sufficient to kill 100% of the cells. Increasing amounts of TNFR1:IgG showed an inhibitory effect on TNF induced apoptosis in WEHI cells (Figure 1) suggesting TNFR1:IgG was able to block TNF activity in vitro. Although not tested in this study, previous studies have demonstrated the efficacy of human TNFR1 on inhibiting rodent TNF-α in animal models of autoimmune diseases [23,24].

### Autoimmune diseases in female NOD

For over two years we have maintained a colony of NOD mice under defined conditions and carefully monitored the onset of both diabetes and change in SG function. Blood glucose levels were monitored starting from 12 weeks of age and the mice were administered daily insulin when they became hyperglycemic (>400 mg/dl). SG activity was monitored by measuring the change in volume of pilocarpine stimulated saliva flow over 20 minutes with respect to BW at 6, 16, 20 and 24 weeks.

**Figure 2:**
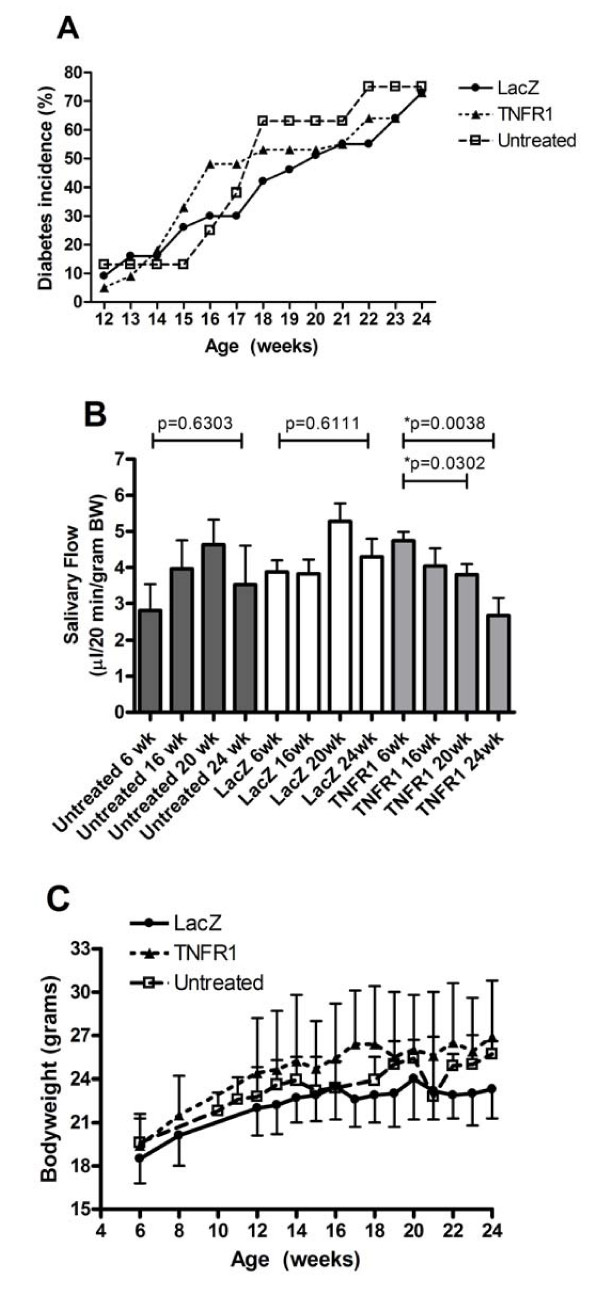
**Diabetes, saliva flow, and body weight in untreated and vector treated groups**. **A) **Blood sugar was monitored starting at week 13 (five weeks post vector delivery) as described in the Materials and Methods section. Data shown are the mean percentage from two independent experiments (N = 8 for untreated, N = 20 for LacZ and N = 19 for TNFR1:IgG). **B) **Saliva was collected as described in the Materials and Methods section over a 20-minute period after stimulation with 0.5 mg/kg BW pilocarpine. Data shown are mean values +/- SEM (N = 8 for untreated, N = 20 for LacZ and N = 19 for TNFR1:IgG). *P*-values are indicated and were determined by non-parametric Wilcoxon's ranksum test. Delivery of TNFR1:IgG resulted in significant decreased saliva flow not seen in control groups. **C) **The weight of the mice (in grams) was measured at the indicate times for LacZ, TNFR1:IgG, and untreated mice. There were no significant changes in bodyweight between all three groups as determined by unpaired student's t-test. Data shown are the mean (+/- SD) of two independent experiments (N = 8 for untreated, N = 11 for LacZ and N = 12 for TNFR1:IgG per experiment).

**Figure 3:**
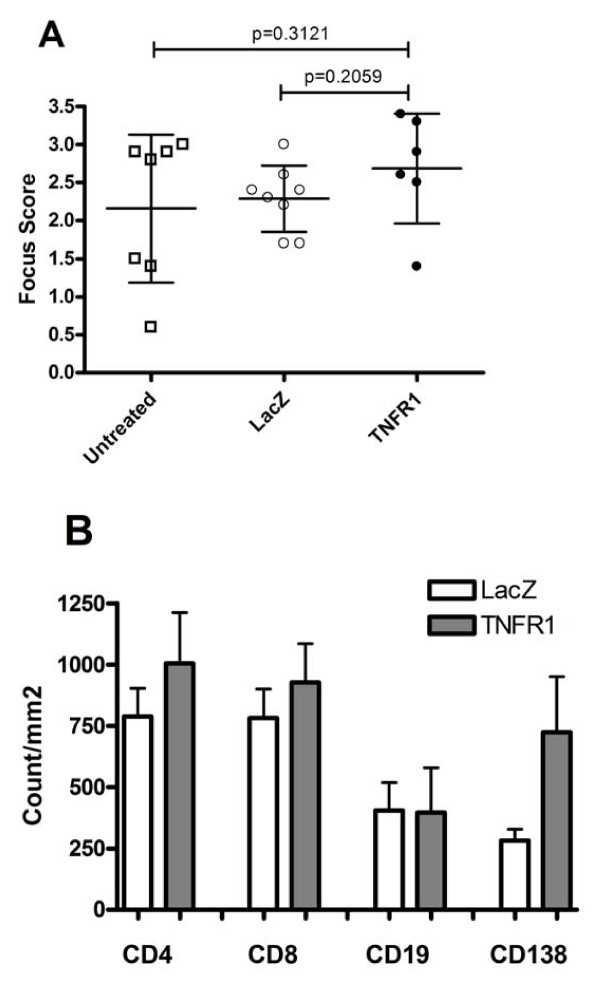
**Focus score and infiltrate analysis in untreated and vector treated groups**. **A) **Salivary glands were removed for histologic analysis at 24 weeks, fixed and stained with Hematoxylin and eosin. Histopathologic assessment was performed and presented as a focus score (see Materials and Methods). Data shown are the mean (+/- SD). No significant change was detected by one-way ANOVA test followed by unpaired student's t-test. Untreated (N = 7), LacZ (N = 8) and TNFR1:IgG (N = 6) mice. **B) **Acetone fixed frozen sections from SGs were analyzed for CD4, CD8, CD19 and CD138 by immunohistochemistry followed by digital analysis as described in the Material and Methods section. Data shown are the mean positive cell counts per mm^2 ^(+/- SEM). Higher means were observed for CD4, CD8 and CD138 in the TNFR1:IgG treated mice, but no significant changes were detected by non-parametric Wilcoxon's ranksum test. LacZ (N = 4) and TNFR1:IgG (N = 8).

The incidence of hyperglycemia increased with age, starting at week 16 (Figure 2A, untreated). At 24 weeks of age, 75% of the mice were hyperglycemic. In contrast, no change in SG activity was detected in these mice (Figure 2B, untreated). Stimulated salivary flow was initiated by subcutaneous injection of 0.5 mg/kg BW pilocarpine and saliva was collected by capillary tube over 20 minutes. At six weeks of age, the mean saliva volume was 2.81 ± 0.72 μl/g BW (mean ± SEM). Mean saliva volume increased over time to 3.96 ± 0.78 and 4.63 ± 0.69 μl/g BW at 16 weeks and 20 weeks respectively. At the end of the study (24 weeks), the mean saliva volume decreased slightly to 3.52 ± 1.08 μl/g BW, but was not lower than the six week value and no statistically significant difference was measured at any time point. Moreover, these mice developed sialadenitis with a mean focus score of 2.2 (Figure 3A). This phenotype has been confirmed over multiple generations of mice with similar results suggesting a stable phenotype in this environment. Furthermore, these measures are independent of the diabetic state of the mouse at these time points.

### TNFR1:IgG expression in the salivary gland does not affect the rate of hyperglycemia or bodyweight

Stable TNFR1:IgG expression in the SGs was achieved by cannulation of the glands and retrograde delivery of the Adeno Associated Virus (AAV) vector encoding either TNFR1:IgG (rAAV2-hTNFR1-mIgG1) or a control vector expressing β-galactosidase (rAAV2-LacZ) to both submandibular SGs of eight-week-old mice (N = 20 mice in the LacZ group and N = 21 mice in the TNFR1:IgG group). Vector DNA was detected by Q-PCR on extracted total DNA from 20-week-old SGs confirming gene transfer (data not shown) and human sTNFR1 was detected in extracted protein confirming protein expression (Table 2). Blood sugar levels were followed starting at 12 weeks of age. In two independent experiments, between 14 weeks and 19 weeks of age the TNFR1:IgG treated group did not show a statistically significant difference in the rate of IDDM compared with the LacZ treated group. At 14 weeks of age, an average of 18% of the animals receiving TNFR1:IgG showed hyperglycemia (blood sugar >400 mg/dl) compared with 16% in the vector control group and by 24 weeks 73% in both vector treated groups had severe hyperglycemia (Figure 2A). This incidence of IDDM was similar to our historic rate in untreated mice (Figure 2A).

**Table 2:** Cytokines in salivary gland extract and plasma

	Salivary gland extract (pg/ml per 20 mg wet SG)			Plasma (pg/ml)		
	
	LacZ	TNFR1:IgG	*P *value	LacZ	TNFR1:IgG	*P *value
mTNF-α	73.0 (53.0)	24.6 (2.5)	0.9273	21.2 (5.3)	45.5 (15.3)	0.3152
MCP-1	325.0 (222.7)	254.6 (70.6)	1.0000	182.0 (49.0)	95.1 (10.8)	0.0727
mIL-4	23.0 (12.2)	3.8 (1.0)	0.0727	1.2 (0.05)	16.1 (2.3)	*0.0061
mIL-5	94.5 (41.4)	7.3 (1.9)	*0.0061	2.3 (0.04)	30.7 (8.3)	0.0727
mIL-6	109.9 (61.4)	42.4 (15.2)	0.3152	20.7 (8.8)	37.0 (5.7)	0.1636
mIFN-γ	657.9 (305.5)	614.9 (216.1)	0.5273	192.9 (28.5)	511.7 (75.0)	*0.0061
mIL-10	37.6 (14.8)	7.7 (2.0)	0.0727	3.0 (0.4)	27.5 (3.3)	*0.0061
mTGF-β1	4342 (1742)	3.0E6 (7.8E5)	*0.0061	7.8E5 (8.1E4)	1840 (187.7)	*0.0061
mIL-12p70	130.2 (54.3)	6.3 (2.3)	*0.0061	18.3 (11.0)	65.8 (7.4)	*0.0242
mIL-17	53.2 (24.3)	5.4 (1.6)	*0.0242	6.4 (3.1)	12.1 (1.8)	0.1636
hTNFR1	0.0 (0.0)	114.4 (43.5)	ND			

Protein expression of immunomodulatory molecules after administration of LacZ and TNFR1:IgG in salivary gland extract and plasma. Data shown are mean values +/- SEM. Significant differences are indicated (*) and were determined by non-parametric Wilcoxon's ranksum test. LacZ (N = 4) and TNFR1:IgG (N = 7). ND = not determined.

Increased disease activity can cause growth retardation; therefore bodyweights were measured in the vector treated groups between 6 and 24 weeks of age and compared to untreated mice. We saw a normal age-dependent increase in bodyweight over time in all three groups, with no significant differences between the groups (Figure 2C). These results suggest that stable TNFR1:IgG expression in the SGs does not affect the rate of hyperglycemia or bodyweight in NOD mice.

### Stimulated saliva flow is inhibited by TNFR1:IgG treatment

To investigate the effect of TNFR1:IgG expression on SG function, stimulated saliva flow was measured at 6, 16, 20 and 24 weeks. Before vector delivery, the mean saliva volume/gram BW was measured in each group. At six weeks of age, the mean volumes for the LacZ (N = 20) and TNFR1:IgG (N = 21) groups were 3.88 ± 0.32 and 4.30 ± 0.49 μl/g BW respectively. After vector delivery, the saliva volumes within the control group followed a similar course observed in our longitudinal studies in untreated mice (Figure 2B). There was no significant difference (*P *= 0.6111) between the mean saliva volume before vector delivery and at 24 weeks (16 weeks post cannulation). In contrast, the saliva volume decreased at all time points in mice receiving TNFR1:IgG compared with the baseline value (4.65 ± 0.32 μl/g BW) prior to cannulation. Salivary flows were 4.04 ± 0.49, 3.80 ± 0.29 and 2.67 ± 0.48 μl/g BW at 16, 20 and 24 weeks, respectively (Figure 2B). Stimulated saliva volumes of animals at 20 and 24 weeks receiving TNFR1:IgG locally in both submandibular glands were significantly decreased compared to their baseline levels (*P *= 0.0302 and *P *= 0.0038 respectively). We were unable to detect any correlation between saliva volumes and blood glucose levels in individual NOD mice (data not shown). These data suggest that expression of a TNF inhibitor in the SGs of NOD mice results in decreased salivary flow.

### The effect of TNFR1:IgG therapy on inflammation in the salivary glands

To determine if this loss of SG activity was the result of autoimmune activity in the glands, we compared the number of focal infiltrations of inflammatory cells within the SGs [25]. Sections from the submandibular glands of TNFR1:IgG (N = 6) and LacZ (N = 8) treated mice sacrificed at 24 weeks were examined histologically with H&E staining to assess inflammatory infiltrates. The focus score (mean ± SD) of mice receiving TNFR1:IgG (2.68 ± 0.42) compared with 2.27 ± 0.43 and 2.17 ± 0.97 for mice receiving LacZ or untreated respectively was slightly increased (Figure 3A). This increase in focus score was not statistically significant when assessed by one-way ANOVA (*P *= 0.4256, not shown). Thus far, our findings suggest no difference in SG activity or histology as a result of AAV vector delivery or LacZ expression which is in agreement with previous studies [15,16]. Therefore, we decided to collect cytokine data from the LacZ vector treated groups to focus on any cytokine changes that were the result of expression of the hTNFR1 study drug.

In addition to scoring the number of foci, infiltrating lymphocytes were phenotyped in both vector treated groups. For both groups, CD4+ and CD8+ T lymphocytes were most prominent followed by B (CD19+) lymphocytes and plasma (CD138+) cells (Figure 3B). Both CD4+ and CD8+ T lymphocytes were slight increased in TNFR1:IgG treated mice with 1004 ± 208 (mean positive cells/mm^2 ^± SEM) and 927 ± 158 respectively compared to mice receiving LacZ (788 ± 115 and 782 ± 117 respectively; Figure 3B). Although with higher variability, the same pattern was seen for plasma cells. Control vector treated mice showed 282 ± 45 plasma cells compared to 724 ± 227 in TNFR1:IgG treated mice. No difference for B lymphocytes was detected between the vector treated groups (Figure 3B). All the differences detected in lymphocytic cell types were not statistically significant when assessed by non-parametric Wilcoxon's ranksum test.

### TNFR1:IgG expression alters cytokine levels in the salivary glands and plasma

**Figure 4:**
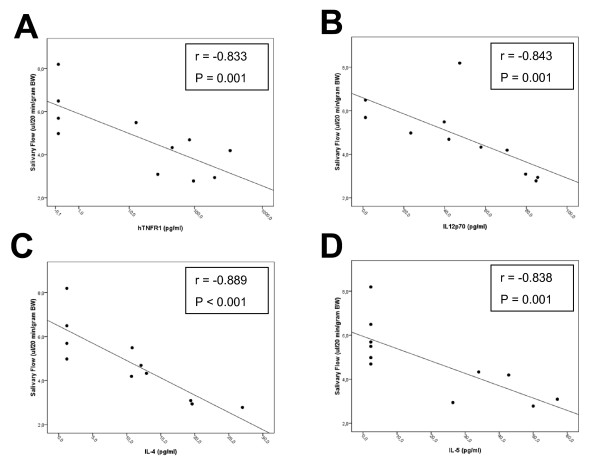
**Correlation cytokines with salivary flow**. Cytokine data were analyzed for correlation with salivary flow from mice at 20 weeks of age using the non-parametric Spearman's Rho test. The correlation coefficient (r) and *P*-values are indicated. Data on the x-axis for figure **A **are plotted in log-scale. Strong correlations were found for hTNFR1 **(A) **in SGs and IL-12p70 **(B)**, IL-4 **(C)**, IL-5 **(D) **in plasma.

To investigate if local expression of TNFR1:IgG in the SG could change cytokine levels either systemically in the plasma or locally in the SGs, plasma samples and SG protein extracts were obtained from mice at 20 weeks (12 weeks post cannulation) and cytokine levels were measured by multiplex analysis (Table 2). Expression of TNFR1:IgG in the SG did not change the level of murine TNF-α, MCP-1, or IL-6 in either plasma or SG samples. In the SG samples, cytokines from the Th1, Th2 and Th17 lineage (mIL-12p70, mIL-5 and mIL-17) were significantly decreased in the TNFR1:IgG SG samples compared with control samples. In contrast, we observed a significant increase in mTGF-β1. No significant change in mIFN-γ or mIL-4 expression in the SG samples from the TNFR1:IgG treated group compared with the control group (Table 2). Further analysis showed a correlation between SG hTNFR1 levels and salivary flow (r = -0.833, *P *= 0.001; Figure 4A). Moreover, SG mTGF-β1, IL-5, IL-12p70 and IL-17 showed also a correlation with salivary flow (r = -0.641, *P *= 0.034; r = 0.604, *P *= 0.049; r = 0.659, *P *= 0.027 and r = 0.604, *P *= 0.049 respectively, data not shown).

Interestingly, many cytokines showed an opposite pattern of expression in the plasma samples compared with the SG samples. Plasma levels of murine IL-4, IL-10, IL-12p70 and IFN-γ were significantly increased and mTGF-β1 was significantly decreased in the TNFR1:IgG treated mice compared with controls (Table 2). IL-5 expression was also increased in plasma TNFR1:IgG treated samples compared with the decrease in the SG sample but the change was not statistically significant. In contrast to the SG samples, IL-17 in the plasma remained unchanged. Also, plasma IL-12p70 showed a correlation with salivary flow (r = -0.843, *P *= 0.001, Figure 4B). Similar results were obtained for plasma IL-4 (r = -0.889, *P *< 0.001, Figure 4C), IL-5 (r = -0.838, *P *= 0.001, Figure 4D), and IFN-γ (r = -0.682, *P *= 0.021, data not shown). These data imply a local effect of TNF-inhibitors, showing increased levels of TGF-β1 and decreased levels of IL-5, IL-12p70 and IL-17 in SGs, accompanied by decreased levels of TGF-β1 and increased levels of IL-4, IFN-γ, and IL-12p70 in plasma. Correlation analysis also suggests that changes in expression of several of these cytokines may be closely linked with changes in salivary gland activity as measured by changes in salivary flow rates (Figure 4).

### The effect of TNFR1:IgG treatment on autoantibody formation

**Figure 5:**
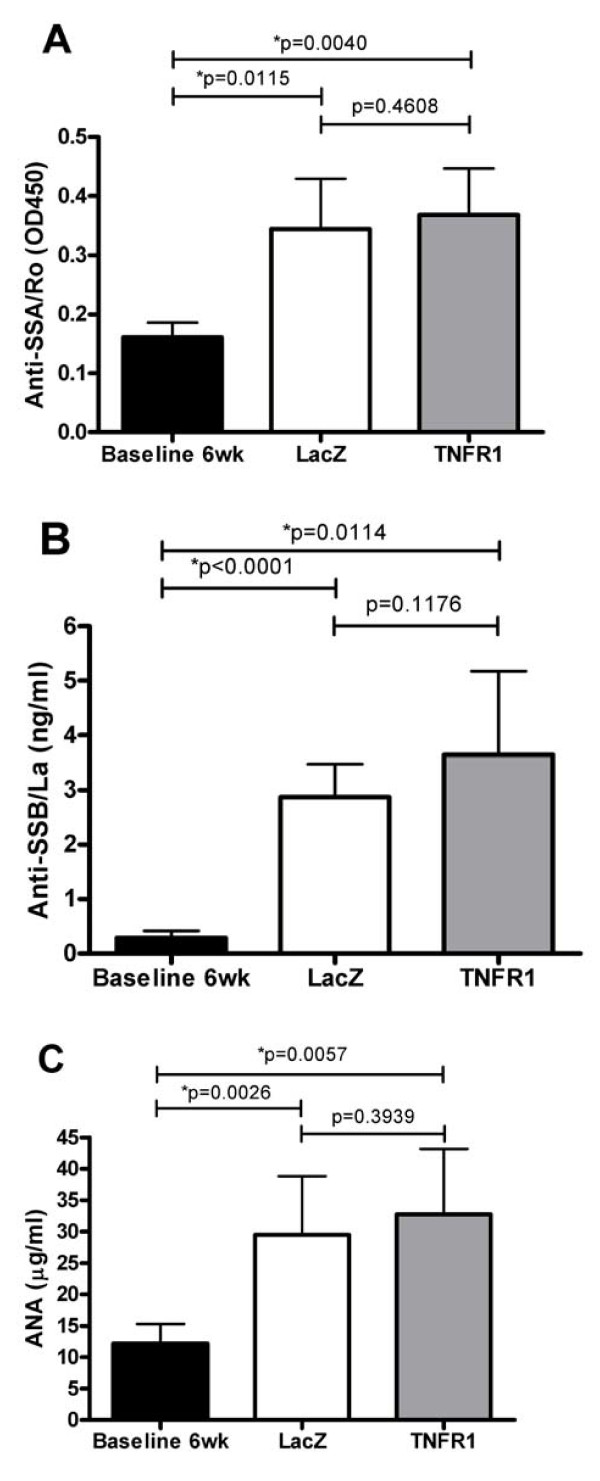
**Autoantibodies in plasma**. Plasma samples were collected and autoantibody levels were measured as described in the Material and Methods section using samples collected at 24 weeks and compared with four different pools of pre-disease six-week-old mice. Antibody levels for SSA/Ro **(A) **SSB/La **(B) **and ANA **(C) **are shown (mean ± SD). Significant differences are indicated (*) and were determined by one-way ANOVA test followed by unpaired student's t-test. LacZ (N = 8) and TNFR1:IgG (N = 6).

Sera from SS patients contain a number of identifiable autoantibodies directed against nuclear, cytoplasmic and cell surface components. Autoantibodies have also been reported to occur in patients receiving anti-TNF therapy and in NOD mice [1,26-28]. Therefore, we compared autoantibody levels in plasma samples from untreated 6 week old mice (N = 4, pooled), 24 weeks old TNFR1:IgG (N = 6) treated and control LacZ (N = 8) treated mice. As shown in Figure 4, autoantibodies did increase with age but there was no clear cut difference between the LacZ and TNFR1:IgG treated groups for anti-SSA antibodies (Figure 5A). There was perhaps a minor trend towards increased levels of anti-SSB (Figure 5B) and ANA (Figure 5C), which did not reach statistical significance. These results imply that characteristic SS autoantibodies are produced over time in NOD mice, but there is no clear cut effect of local TNF blockade on the autoantibody profile.

## Discussion

In order to better understand the local role of TNF and the effect of TNF soluble receptor based therapies on SG activity, we have stably expressed soluble TNFR1:IgG fusion protein locally in the SGs of NOD mice and followed stimulated saliva flow, sialadenitis, cytokine production, autoantibody levels, and insulin-dependent diabetes mellitus (IDDM) over 18 weeks. This local therapy resulted in decreased stimulated saliva flow and reduced Th1, Th2 and Th17 cytokine levels in the SG accompanied by increased levels of Th1 and Th2 cytokines in plasma.

The reduced SG activity after local TNF blockade suggests a protective role for TNF in SG function. TNF has been reported to exhibit anti-inflammatory activities, for instance by blocking the development of autoreactive T cells or by altering the balance of regulatory T cells [10,11]. Recently, the anti-inflammatory effects of TNF were reported to be mediated through TNFR2 in tolerogenic TGF-β treated antigen presenting cells (APC) and blocking of TNFR1 signaling enhanced the ability of APCs to secrete TGF-β in response to TGF-β exposure [29]. Consistent with this notion, TNFR1:IgG treatment resulted in increased TGF-β1 tissue levels. The unaltered IFN-γ and MCP-1 levels suggest an IFN-γ independent pathway. In plasma we found an opposite pattern after TNFR1:IgG treatment, with increased levels of IL-4, IL-12p70 and, IFN-γ and decreased expression of TGF-β1. Of interest, IL-12p70 (together with IFN-γ) has been reported to be elevated in serum of SS patients [30]. Moreover, recently we have reported salivary dysfunction in IL-12 transgenic mice, providing a new model of SS [31]. The systemic decrease of TGF-β1 levels found in the present study is in agreement with the SS-like lymphoproliferation seen in TGF-β KO mice [32]. Our data indicate that systemic upregulation of IL-12p70 and downregulation of TGF-β1 may play an important role in SS and these cytokines might promote SG dysfunction after anti-TNF treatment. The change in SG function after TNFR1:IgG treatment is in line with previous observations showing that under certain circumstances patients on anti-TNF therapy may develop additional autoimmune complications (for example, demyelinating events) [33-36]. TNF blockers also have been shown to induce antinuclear antibodies (ANA) and antibodies directed against double stranded (ds) DNA in autoimmune diseases, such as RA, spondyloarthritis (SpA) and systemic lupus erythematosus (SLE) [27,28].

There was no statistically significant change in focal infiltration of the gland or autoantibody levels in our study. Similarly, we previously found that SG activity can be affected by the local expression of immunomodulatory proteins independent of the focus score [16]. Additionally, phenotyping of the infiltrating lymphocytes showed a trend to increased positive cell counts for plasma cells, CD4+ and CD8+ T lymphocytes in TNFR1:IgG treated SGs. Our data suggest that i) expression of TNFR1:IgG does not impair the ability of lymphocytes to migrate to areas of inflammation and ii) there is no clear cut correlation between decreased saliva flow and the severity of inflammation within the glands. A large body of literature has determined that in rodents, AAV2 based vectors only trigger a minimal and largely transient immune response. Although, we have not specifically tested for the generation of anti-AAV2 antibodies or cytotoxic T lymphocytes (CTLs) in this study, the SG activity and infiltrate scores were very similar for both untreated mice and those cannulated with AAV2 LacZ vector, suggesting the AAV2 vector itself has minimal response. In other studies we have also used a vector that does not encode a transgene such as LacZ and found similar results (data not shown).

There is at present no generally accepted animal model of SS, which represents all the features of this condition. However, in addition to its utility in studying IDDM, the NOD mouse can also develop other autoimmune conditions such as gender- and age-specific mononuclear gland infiltrates and exocrine dysfunction of salivary and lacrimal glands that resemble Sjögren's syndrome [37]. We have previously used this model successfully to demonstrate the stimulatory effect of IL-10 [15] and vasoactive intestinal peptide gene transfer [16] on SG activity. The development of autoimmune disease in these mice can, however, be affected by environmental and experimental conditions and is also dependent on the specific substrain of the NOD mice [38-40]. Over the last two years we have maintained a colony of NOD mice under defined conditions that develop sialadenitis. Consistent with our recent observations [38], the mice developed SG infiltrates and autoantibodies without developing a spontaneous loss of SG activity. Also, other NOD-derived strains have been reported as SS animal models, such as NOD.B10-H2b [41] and C57BL/6.NOD-Aec1 Aec2 [42]. Each of these strains has advantages compared with NOD/LTJ mice and as they become more widely studied, may be useful models for examining the long term effects of immunomodulatory proteins which is difficult in NOD/LTJ mice. The results presented here also show that local TNFR1:IgG treatment administered to the SG does not affect the incidence of diabetes in this model. Several studies have indicated that systemically increasing TNF levels can prevent the onset of IDDM in NOD mice [11,43]. Potential mechanisms include the effect of TNF on signal transduction through the T-Cell Receptor (TCR), including NF-κB expression, and/or a direct effect of TNF on T-cell apoptosis in the periphery [11,44]. On the other hand, systemic TNFR1:IgG treatment prevented diabetes in NOD mice [45]. Taken together, the role of TNF in the pathogenesis of diabetes in NOD mice is still controversial [43]. Conceivably, the systemic levels of TNFR1:IgG in our study were too low to influence the disease proves in the pancreas. In contrast to TNFR2, the type 1 TNF receptor can bind both TNF-α and lymphotoxin (LT)-α. The results shown in our experiments are likely the result of binding both cytokines. At this point, we are not able to distinguish between both effects and therefore cannot conclude that the effect on gland activity is caused by blocking TNF-α alone. Further mechanistic experiments are needed to explain the exact role of anti-TNF in SS.

## Conclusions

The data presented here do not support the notion that local TNF blockade may have a beneficial effect in SS. In contrast, TNF blockade might worsen SG function in this disease.

## Abbreviations

ANA: antinuclear antibody; APC: antigen presenting cell; BSA: bovine serum albumin; BW: bodyweight; CMV: cytomegalovirus; CsCl: cesium chloride; CTL: cytotoxic T lymphocyte; FBS: fetal bovine serum; H&E: hematoxylin & eosin; HRP: horseradish peroxidase; IDDM: insulin-dependent diabetes mellitus; IFN-γ: interferon-γ; IL: interleukin; IM: intramuscular; ITR: inverted terminal repeat; LT-α: lymphotoxin-α; MCP: monocyte chemoattractant protein; MNV: mouse norovirus; MPV: mouse parvovirus; MVM: minute virus of mice; NOD: non obese diabetic; PBS: phosphate buffered saline; Q-PCR: quantitative-polymerase chain reaction; rAAV: recombinant adeno associated virus; RA: rheumatoid arthritis; SC: subcutaneous; SG: salivary gland; SpA: spondyloarthritis; SS: Sjögren's syndrome; TCR: T cell receptor; TGF-β: tumor growth factor-β; TNF: tumor necrosis factor; TNFR1:IgG: tumor necrosis factor receptor type 1 coupled to the Fc part of Immunoglobulin G; VIP: vasoactive intestinal peptide; WEHI: human fibrosarcoma cells.

## Competing interests

The authors declare that they have no competing interests.

## Authors' contributions

JLV and JAC designed the study. JLV, HY and NR acquired the data. JLV, HY, PPT and JAC analyzed, interpreted and evaluated the data. JLV, MRK, PPT and JAC prepared the manuscript.
